# The Use of Dynamic Tracer Concentration in Veins for Quantitative DCE-MRI Kinetic Analysis in Head and Neck

**DOI:** 10.1371/journal.pone.0059885

**Published:** 2013-03-20

**Authors:** Jing Yuan, Steven Kwok Keung Chow, Qinwei Zhang, David Ka Wai Yeung, Anil T. Ahuja, Ann D. King

**Affiliations:** 1 Department of Imaging and Interventional Radiology, The Chinese University of Hong Kong, Shatin, New Territories, Hong Kong, China; 2 CUHK Shenzhen Research Institute, Shenzhen, Guangdong, China; University of Maryland, United States of America

## Abstract

**Background:**

Head and neck Magnetic Resonance (MR) Images are vulnerable to the arterial blood in-flow effect. To compensate for this effect and enhance accuracy and reproducibility, dynamic tracer concentration in veins was proposed and investigated for quantitative dynamic contrast-enhanced (DCE) MRI analysis in head and neck.

**Methodology:**

21 patients with head and neck tumors underwent DCE-MRI at 3T. An automated method was developed for blood vessel selection and separation. Dynamic concentration-time-curves (CTCs) in arteries and veins were used for the Tofts model parameter estimations. The estimation differences by using CTCs in arteries and veins were compared. Artery and vein voxels were accurately separated by the automated method. Remarkable inter-slice tracer concentration differences were found in arteries while the inter-slice concentration differences in veins were moderate. Tofts model fitting by using the CTCs in arteries and veins produced significantly different parameter estimations. The individual artery CTCs resulted in large (>50% generally) inter-slice parameter estimation variations. Better inter-slice consistency was achieved by using the vein CTCs.

**Conclusions:**

The use of vein CTCs helps to compensate for arterial in-flow effect and reduce kinetic parameter estimation error and inconsistency for head and neck DCE-MRI.

## Introduction

Angiogenesis is vital for the growth and metastasis of malignant tumors. Dynamic contrast-enhanced (DCE) MRI is a valuable non-invasive imaging method that enables the investigation of tissue microvascular environment for many clinical oncology applications. To conduct DCE-MRI, paramagnetic gadolinium based contrast agent, also called tracer, is administrated and passes through the capillary bed where it leaks into the extravascular-extracellular space (EES). This leakage process is influenced by factors like the blood flow, permeability and surface area of the microvessels as well as the volume of EES. The accumulation of tracer concentration in the EES induces longitudinal relaxation time (T1) shortening effect and leads to image intensity enhancement when acquired by T1-weighted MRI pulse sequences. By dynamically monitoring the MR signal variation with time before and after tracer administration, information on the microvascular structure and function could be derived through the subsequent pharmacokinetic analysis on the dynamic image series. DCE-MRI is now playing a more important role in clinical oncology applications such as cancer characterization [1], treatment evaluation [2] and development of anti-angiogenic and vascular-targeting drugs [3], including for those cancers that are located in head and neck [4]–[10]. Although DCE-MRI could be analyzed semi-quantitatively to derive heuristic parameters like maximum enhancement intensity and rate, and the area under dynamic curve, quantitative DCE-MRI analysis based on pharmacokinetic models [11], [12] is able to retrieve physiologically relevant parameters that are independent of scanner and imaging protocols, and hence holds the potentials for standardized multi-center data comparison and clinical trial study.

For most pharmacokinetic models, the measurement of arterial input function (AIF), i.e. the dynamic tracer concentration in the arterial blood plasma, is essential. The accurate measurement of the AIF remains one of the major challenges that hamper the reproducibility of DCE-MRI kinetic analysis and the widespread acceptance of DCE-MRI into clinical practice [13]–[15]. AIF is a complicated function which is influenced by many technical factors (including spatial and temporal resolution, dose and rate of tracer injection, accuracy of the T1 measurements, in-flow effect, partial volume effect, and B1 inhomogeneity [16]–[18]) and many patient-related factors (including heart output rate, vascular tone, hematocrit, tracer distribution in the body and kidney function). In the head and neck there are additional patient-related factors that are impediments to accurate AIF measurement. These factors include tissue susceptibility effects, motion artifacts due to blood pulsation and swallowing, and vascular disease such as atherosclerosis which may cause narrowing and turbulent flow in the artery.

This study proposes to overcome some of the difficulties of measuring the AIF by measuring the dynamic tracer concentration in the jugular veins rather than in the carotid arteries, in order to alleviate the influence of in-flow effect and provide a larger caliber vessel which is less prone to disease for head and neck DCE-MRI analysis. The tracer concentration measurement in veins has been adopted in some brain perfusion studies, where the measurement of venous output function (VOF) in the superior sagittal sinus [19], [20] has been used to reduce partial volume effect, but to the best of our knowledge veins have not been utilized in the neck.

In DCE-MRI, in-flow effect manifests as a signal enhancement due to the flow of fully-polarized fresh blood into the imaging volume and results in both a strong increase in the absolute signal intensity and a significant reduction in the dynamic range of contrast enhancement [21]. Moreover, in-flow effect also induces the errors for *in vivo* blood T1 measurement and hence is considered as a major confounding factor of the AIF measurement [17]. In-flow effect is dependent on many factors, including the tracer concentration, blood flow velocity, slice location, as well as imaging parameters like flip angle, TE and TR, so is difficult to be precisely modeled and completely compensated. Advanced in-flow correction methods have been proposed [21], [22], however, they are still rarely applied in DCE-MRI data acquisition due to the extra scan time, the complicated calibration, and the inherent unsteadiness of blood flow in arteries.

Blood plasma could be considered as a single pool for high velocity flows with low permeability between the plasma and the extra-vascular space [12]. The major organ between the arterial and venous circulation in the head and neck is the brain, which as a result of the blood brain barrier (BBB) has a low permeability. In addition the blood travel time between carotid arteries and jugular veins is short. Therefore, tracer concentration in jugular veins and carotid arteries is assumed to be nearly equal within this short time delay. Upon this presumption, the use of contrast agent concentration in the jugular veins could greatly alleviate the in-flow effect in that the blood flow velocity in the jugular veins (18±3 cm/s) [23] is much slower than that in the carotid arteries (maximum systolic 108.2±3.8 cm/s, cycle-averaged 38.8±1.5 cm/s) [24]. Meanwhile, blood in the jugular veins flows more steadily during the heart cycle with less pulsation. Unlike population-averaged AIF [25], this method allows the direct measurement on each individual subject and respects the underlying inter-individual variation in physiology and the tracer injection protocol differences. Compared to other approaches, this method is convenient and the measurement accuracy of tracer concentration in the vein is robust to slice locations and less susceptible to T1 measurement errors. Compared to some advanced in-flow correction methods [21], [22], no complicated calibration and correction algorithm and extra scan time are required either. Therefore measurements obtained from the jugular vein could be applied quite straightforwardly using the existing kinetic DCE-MRI analysis tools by clinicians and potentially. This method would benefit multicenter cross-sectional studies by standardizing the AIF measurement in head and neck after validation by more clinical studies.

## Results

Primary tumors were identified from anatomical TSE images and their sites were supraglottic (n = 6), oropharynx (n = 9), hypopharynx (n = 5), and buccal mucosa (n = 1). Primary tumor sizes ranged in maximum axial diameter from 10 mm to 52 mm (mean = 37 mm). The corresponding areas ranged from 18 mm^2^ to 1123 mm^2^ (mean = 456 mm^2^). Twelve metastatic nodes were also identified with sizes ranged in minimum axial diameter from 15 mm to 37 mm, mean = 27 mm (area from 219 mm^2^ to 1165 mm^2^, mean = 583 mm^2^).

**Figure 1:**
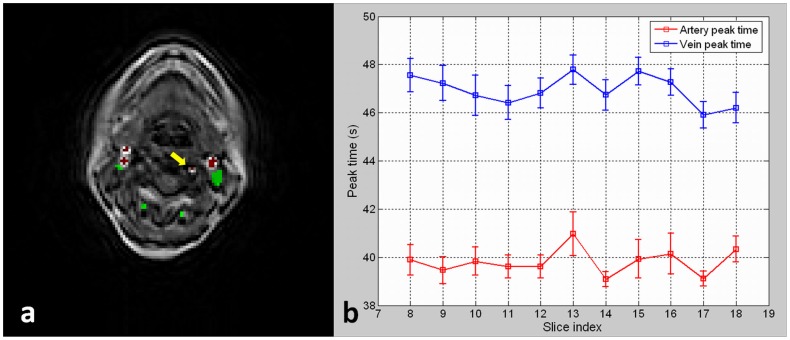
Blood Vessel Extraction and Labeling. (a) Arteries and veins labeled by the automated vessel extraction method. The isolated vertebral artery voxel (the yellow arrow) was excluded from the vessel extraction due to its proneness to partial volume effect. (b) The dynamic peak times for artery and vein voxels in image slices. The average peak time of arteries appeared around 7.5 s earlier than veins.

Arteries and veins were well separated by the automated method in all patients and manually confirmed by the radiologist. Figure 1a shows the labeling of arteries and veins on an image slice of one patient, in which red spots labels artery voxels and green spots labels vein voxels. Boundary voxels around vessels were excluded automatically to remove the partial volume effect. The isolated vertebral artery voxel (yellow arrow), was also excluded due to its proneness to partial volume effect. For all patients, the result showed that artery voxels primarily came from the carotid arteries and vein voxels primarily came from the jugular veins because of their large cross section areas. Figure 1b shows the dynamic peak times for artery and vein voxels in the image slices. The square marker denoted the average peak time value and the error bar denoted the standard deviation of the peak time for artery and vein voxels for each slice. It is shown that the average peak time of arteries appeared about 7.5 s earlier than veins. The peak times and the time delays in arteries and veins were stable for slices.

**Figure 2:**
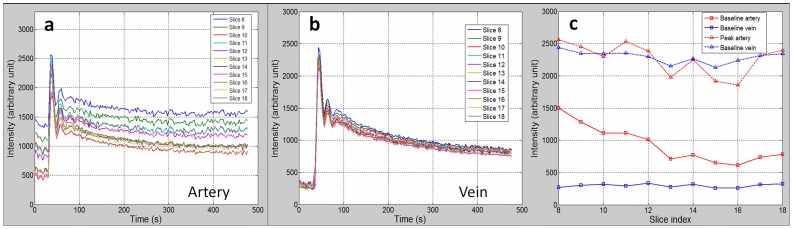
Inter-slice Variations of Intensity-time-curves in Arteries and Veins. (a) The dynamic intensity-time-curves (ITCs) in artery voxels for image slices. Severe in-flow effect resulted in the inter-slice ITC differences. (b) The dynamic ITCs in vein voxels for image slices. Inter-slice ITC differences were moderate due to the reduced in-flow effect in veins. (c) The baseline and peak intensities in arteries and veins for image slices. The baseline intensity in arteries decreased by a factor of two with the ascending image slices.

The averaged dynamic ITCs for artery voxels and vein voxels for each image slice were shown in Fig. 2a and 2b respectively. Pronounced inter-slice differences in baseline intensity, peak intensity and wash-out level were found for artery voxels, while the dynamic ITCs for vein voxels were quite consistent between slices. Figure 2c illustrates the baseline and peak intensities in arteries and veins. The baseline intensity in arteries decreased approximately by a factor of two from the most inferior slice 8 to the superior slice 18. In contrast, the baseline intensity remained quite stable in veins within all slices.

**Figure 3:**
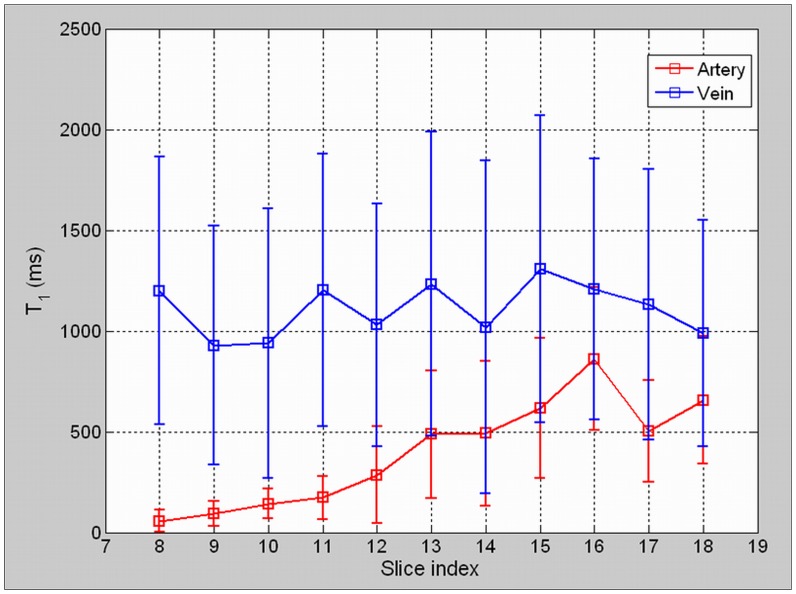
The Measured T1 Values in Artery and Vein by using the Dual-Flip-Angle Method. T1 values in the artery voxels increased with the ascending slices, but were significantly underestimated for all image slices. As comparison, the measured T1 values in vein voxels were relatively stable, whereas were moderately underestimated as well.

The blood in-flow effect on the measured T1 values for artery and vein voxels were illustrated in Fig. 3, which also explain the motivation of using literature values of blood T1 for analysis. Measured T1 values in the artery voxels increased with the ascending slices. For vein voxels, T1 values were relatively stable (mean 1120 ms) between slices. The in-flow effect severely compromised the T1 measurement accuracy in arteries, while its effect on venous blood T1 measurement was relatively moderate. The large standard deviation of T1 measurement could be explained by partial volume effect, non-uniform intra-vessel blood flow speed, as well as the dual-flip-angle limitation.

**Figure 4:**
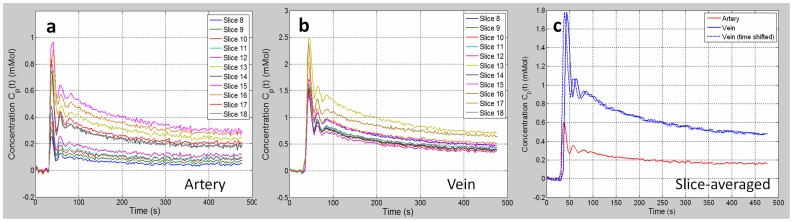
Inter-slice Variations of Consentration-time-curves in Arteries and Veins. (a) The dynamic concentration-time curves (CTCs) in arteries for image slices. The remarkable inter-slice concentration consistency was resulted by the severe in-flow effect. (b) Relatively consistent dynamic CTCs in veins. (c) The averaged dynamic CTCs in arteries and veins along with the time shifted CTC in veins with the peak time aligned to the peak time in arteries. The tracer concentration in arteries may be significantly underestimated due to the in-flow effect.

Large variability of dynamic concentration in arteries (based on ITCs in Fig. 2) was found between slices even if the constant literature T1 value was used, as shown in Fig. 4a. As comparison, relatively consistent dynamic CTCs were achieved in the vein voxels (Fig. 4b). The slice-averaged dynamic CTCs in arteries and veins were shown in Fig. 4c along with the time shifted CTC in veins with the peak time aligned to the peak time in arteries. The peak concentration of the averaged vein CTC was about three times higher than that of artery CTC, which was underestimated due to the fast arterial blood flow.

**Figure 5:**
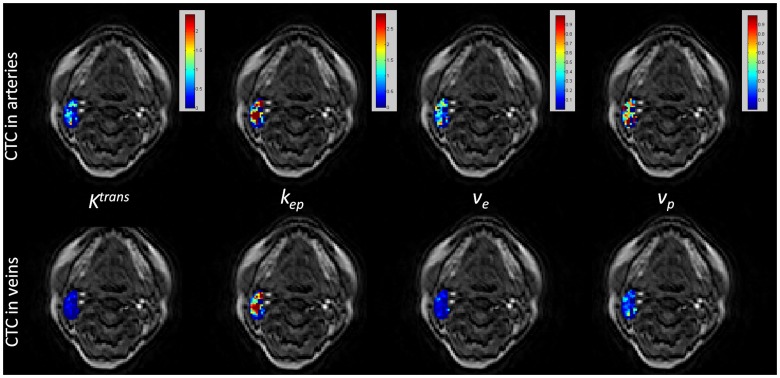
Kinetic Parameter Maps within a Metastatic Node. The kinetic parameter maps (goodness of fit R^2^> = 0.8) within a metastatic node overlaid on the first time point DCE image by using the slice-averaged CTCs in arteries (first row) and veins (second row).

For voxel-wise fitting within the lesion ROIs, around 7–24% of total voxels for different subjects failed of fitting due to the violation of fitting restrictions or the poor goodness of fit when arterial CTCs were used, while the corresponding voxel fraction was reduced to 3–15% by using venous CTCs. The successfully fitted pixels had the average goodness of fit R^2^ of 0.84 and 0.89 for all lesions by using arterial and venous CTCs, respectively. These results indicated that the Tofts model fitting by using venous CTCs was more robust and less vulnerable to the noise and signal fluctuations. Representative Tofts parameter maps within a metastatic node ROI (overlaid on the first time point pre-contrast DCE image) by using the slice-averaged dynamic CTCs in arteries and veins were illustrated in Fig. 5.

**Figure 6:**
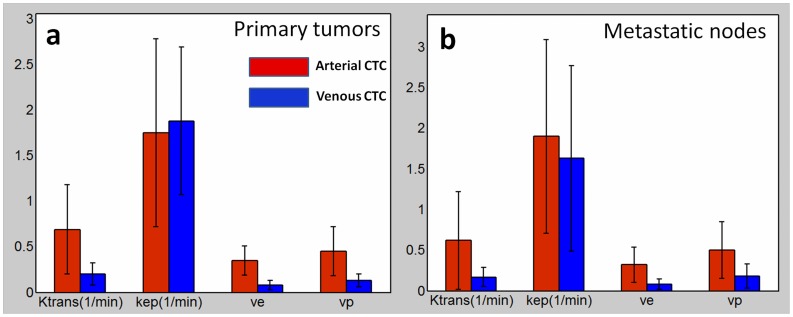
Comparison of Fitting Result in Primary Tumors and Metastatic Nodes. The Tofts model fitting results by using the slice-averaged dynamic CTCs in arteries and veins in primary tumors (a) and metastatic nodes (b). Except for k_ep_, significant differences were found in the estimations of K^trans^, v_e_ and v_p_ by using the dynamic CTCs in arteries and veins.

The fitting results by using the slice-averaged dynamic CTCs in arteries and veins were compared by bar plots for primary tumors (n = 21) in Fig. 6a and for metastatic nodes (n = 12) in Fig. 6b. All pharmacokinetic parameters were expressed as slice and voxel averaged values within the primary tumor and metastatic node ROIs by the bar heights in Fig. 6. Except for k_ep_ (p = 0.081 for primary tumors, p = 0.093 for metastatic nodes, paired student’s t-test), significant differences were found in the estimated K^trans^, v_e_ and v_p_ by using the dynamic CTCs in arteries and veins (p<0.01 for both primary tumors and metastatic nodes, paired student’s t-test). The estimated K^trans^, v_e_ and v_p_ by the use of dynamic CTCs in arteries were generally around 3–3.5 times larger than the corresponding estimations by the use of CTCs in veins.

**Figure 7:**
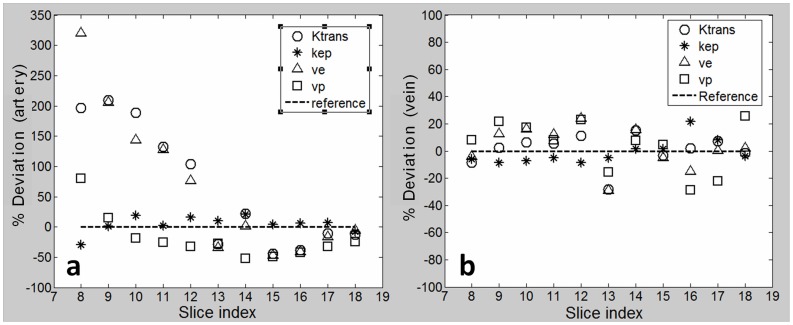
Comparison of Inter-slice Variation of Tofts Fitting Results on A Primary Tumor. Tofts model fittings on a primary tumor by using the individually extracted CTCs from each slice compared to the fitting results by using the slice-averaged CTCs as a reference. (a) Large deviations over 50% were found in the estimations of K^trans^, v_e_ and v_p_ by the use of individual artery CTCs from the interior slices (slices 8–12). (b) Relatively stable and consistent fitting results (<35% deviation) were achieved by using the individual CTCs in veins.

Tofts parameter fitting results (slice and voxel averaged value) on a primary tumor by using individually extracted CTCs for each slice (Fig. 4a and 4b) were compared to the fitting results by using the averaged CTCs (Fig. 4c). Fig. 7a shows the percent deviations induced by the use of the extracted CTCs in arteries for each slice relative to the reference of the fitting results using the slice-averaged CTC in arteries. The fitting results considerably varied compared to the reference. Large deviations over 50% were found in the estimations of K^trans^, v_e_ and v_p_, especially by the use of CTCs from the inferior slices (slices 8–12). ANOVA analysis showed that K^trans^, v_e_ and v_p_ derived from the individual CTCs of each slice were generally significantly different (p<0.01) from the reference (except for a smaller number of cases such as v_p_ derived from the CTC of slice 9 and v_e_ derived from the CTC of slice 14), while k_ep_ showed no significant difference. As comparison, the fitting results by using the individual CTCs in veins (Fig. 7b) were much more stable and consistent compared to the reference by using the slice-averaged CTC in veins. The fitting deviations were all smaller than 35% for estimations of all physiological parameters. Generally, no significant differences in kinetic parameter estimation were found by the use of vein CTCs from each individual slice compared to the reference except for a small number of cases, for example, K^trans^ and v_e_ derived by the CTC of slice 13, which may be attributed to the relatively low SNR of the images or the tissue heterogeneities.

## Discussion

In physiology, arteries are usually the feeding vessels to capillaries and the dynamic tracer concentration in arteries should be used for pharmacokinetic modeling. In opposition, veins are usually blood draining vessels from capillaries that reflect blood outflow from tissues. However, the dynamic tracer concentration in veins can be considered equal to that in arteries for a single blood-pool and low permeability model between the plasma and the extra-vascular space. For head-and-neck DCE-MRI, this assumption can be satisfied because the major tissue, brain, in between the blood flow pathway normally has low permeability. Meanwhile, the transport of tracer out of the vasculature and hence the depletion of intravascular concentration may also be negligible due to the small tracer bolus arrival time difference in carotid arteries and jugular veins. However, the depletion of intravascular concentration is still difficult to be validated by clinical means and should be further investigated in the future. It is also worth pointing out that the applicability of the proposed method may not be generalized to other tissues like liver, and to patients with compromised BBB, where the shape of the venous CTC can be substantially modulated by contrast agent exchange along the blood flow pathway. The presented approach could also introduce some insecurities such as a variable transit time through the brain. In particular, intracranial masses such as metastases or meningeomas or sinus thrombosis may affect the venous outflow. Under these conditions, the reliability of the venous CTC may be compromised and has to be further addressed in future studies.

It is worth noting that the measured much shorter of T1 in arteries primarily attributed to the in-flow effect rather than the uncertainty induced by the dual-flip-angle method [16], [17]. The adoption of literature arterial blood T1 values of 1550 ms might yield the under-estimation of AIF. However, Tofts parameter estimation deviation induced by the limitation of dual-flip-angle method [26], [27] would not result in biased physiological parameter estimation comparison between using the dynamic tracer concentration in arteries and veins.

Given the high temporal resolution of 2.59 s in this study, not only time shift between arterial and venous concentrations, but also contrast agent dispersion could be observed, reflected by a slower increase to peak for venous concentration curves (Fig. 4c). However, the broadening of the first bolus peak in veins by dispersion was observed small due to the low tissue permeability along the blood flow pathway and the short delay of bolus arrival. In spite of this small dispersion, its effect on kinetic parameter estimation should be further investigated in future studies.

As seen in Fig. 4, there were still noticeable inter-slice differences of venous CTC curves (Fig. 4b), although much smaller than those of arterial CTC curves (Fig. 4a). This could be partially explained by the presence of venous blood flow. Although its velocity is much slower than that in arteries, it could still induce similar (but smaller) variability as in AIF extraction. In this respect, similar flow correction methods employed for AIF extraction may also be applicable for the further reduction of venous CTC variations.

The use of the averaged CTCs in arteries and veins for Tofts model generated remarkably different parameter estimation results (Fig. 6) except for k_ep_. The similarity in k_ep_ estimation was because that k_ep_ was primarily determined by the tissue CTC shape (k_ep_ only appears inside the integral item, which determines the dynamic curve shape, as an independent parameter to be fit in Eq. 2) rather than the absolute concentration values according to the Tofts modeling [12]. As seen in Fig. 4, the slice-averaged arterial and venous CTC after time shift had very similar shape pattern but just majorly differed on the absolute concentration value, so it was expectable that their derived k_ep_ values should be close. It was interesting to find that K^trans^ and v_e_ values derived from artery CTC were closer to the literature reported values in the literature [10]. This observation could be explained since the method in this literature was quite similar to our method by using the artery CTC, without accounting for in-flow effect in arteries. However, we considered that the parameter estimation using the vein CTC should be more reliable due to the lower in-flow contamination.

This study only investigated the DCE-MRI quantification at a fixed tracer dose administration of 0.1 mmol/kg of body weight. This dose is recommended by the Imaging Committee of the Experimental Cancer Medicine Centres (ECMC) for the standardization of DCE-MRI acquisition and quantification [28]. In principle, pharmacokinetic parametric mapping should be independent of tracer dose. In practice, the dose of tracer may affect DCE-MRI signal enhancement level and hence slightly affect the accuracy and uncertainty of quantification results. The report on the optimized tracer dose for better DCE-MRI quantification is still sparse [29] and may need to be further explored. The lack of reproducibility study is another limitation of this study due to the scope of the current ethical approval. The comparison of reproducibility of pharmacokinetic quantification by using arterial and venous CTC is of importance and needs to be conducted in the future. Nonetheless, the current study showed that the goodness of fit by using the venous CTC was better than that by using the arterial CTC, indicating the advantage of more reliable and robust fitting in mathematics and less proneness to noise and signal fluctuations by using the venous CTC.

In conclusion, the use of dynamic tracer concentration in veins for quantitative DCE-MRI kinetic analysis in head and neck was proposed and presented to compensate for the severe in-flow effect caused by the fast and pulsatile blood flow in arteries. More reliable and consistent parameter estimations based on Tofts model could be achieved by using the vein CTCs compared to artery CTCs.

## Materials and Methods

### Ethics Statement

Ethical review board approval was obtained for DCE-MRI exam and data analysis.

### Patients and MRI Experiment

MR imaging was performed on 21 patients (3 females, 18 males, mean age 57.5 years) with untreated HNSCC who had no previous history of a head and neck cancer. Informed consent was obtained before the DCE-MRI examination.

All DCE-MRI scans were performed on a 3T Philips Achieva MRI scanner (Philips Medical Systems, Best, The Netherlands) using a 3D spoiled gradient echo sequence. Body coil was used for excitation and a 16-channel head and neck array coil was used as signal receiver. Imaging parameters for DCE-MRI included: TR/TE = 3.9 ms/0.9 ms, flip angle = 15°, FOV = 230 mm×230 mm×100 mm, matrix = 128×128×25, voxel size = 1.8 mm×1.8 mm×4 mm, SENSE factor = 4. 185 dynamic images for each slice were acquired at a temporal resolution of 2.59 seconds with a total DCE-MRI scan time of around eight minutes. Contrast agent (CA) injection was performed in the form of a bolus injection of Gd-DOTA (Dotarem, Guerbet, France) at a concentration of 0.1 mmol/kg of body weight, using a power injector pump (Medrad, Pittsburgh, Pa) through a 21-gauge intravenous catheter in the right antecubital vein. The injection rate was set at 2.5 mL/s, followed by a 20-ml saline flush at the same injection rate. The CA injection started at six seconds after the commencement of dynamic image acquisitions. Prior to the dynamic image acquisitions, a baseline image for each slice was acquired with the identical imaging parameters as DCE acquisition except a flip angle of 7° was used. T1 maps were calculated from the baseline images and the pre-contrast images using the dual-flip-angle method (7° and 15°) [30]. Immediately after the DCE-MRI scan, anatomical images were acquired as part of our routine clinical scan using a 3D turbo spin echo (TSE) sequence (TE/TR = 10 ms/620 ms, Turbo factor = 4, FOV = 230 mm×180 mm×200 mm, voxel size = 0.55 mm×0.79 mm×4 mm, SENSE factor = 1.5, number of signal average NSA = 2).

### DCE-MRI Analysis

DCE-MRI dynamic images were exported and processed off-line using in-house Matlab (The MathWorks, Natick, MA, USA) programs.

Only the central eleven inner slices within the imaging slab were used for vessel extraction to remove the inhomogeneous inter-slice B1 excitation profile for outer slices. An automated vessel voxel extraction method was used [31]–[33] and briefly described here. The average maximum dynamic intensities for all voxels (S_max-avrg_) except for the background were calculated. Then, a voxel was labeled as a vessel (artery or vein) voxel if its maximum intensity was greater than S_max-avg_ plus three times the standard deviation (SD) of S_max-avrg_. Artery voxels were separated from vein voxels according to the time of the maximum intensity (peak time). The isolated single vessel voxels were removed because they were prone to partial volume corruption. The average signal intensities of the artery and vein voxels were calculated to create the signal ITCs. Signal ITCs were then converted into the plasma concentration C_p_(t) according to
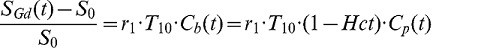
(1)where S_Gd_(t) and S_0_ denote post-contrast image intensity at time *t* and pre-contrast baseline image intensity, respectively. r_1_ is the tracer relaxivity (∼4.5 s^−1^ mM^−1^). T_10_ is the pre-contrast intrinsic T1 relaxation time. C_b_(t) and C_p_(t) denote the tracer concentration in blood and plasma respectively. *Hct* is hematocrit and assumed to be 0.42.

Extended Tofts model was applied for estimation of physiological parameters [25], [34]

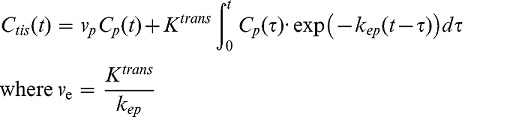
(2)where 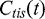 is the concentration of contrast agent in the tissue at time *t*, estimated by the relative signal enhancement method similar to Eq. 1 and 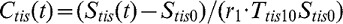. K^trans^, k_ep_, v_e_, and v_p_ denote volume transfer constant, rate constant between blood plasma and extravascular-extracellular space, the volume fraction of EES and volume fraction of plasma, respectively.

To compensate the in-flow induced T1 underestimation and the measured inter-slice T1 variation [17], [21], literature T1 values of 1550 ms [35] for arterial blood and 1852 ms [23] for venous blood at 3T were adopted for the conversion of blood ITCs to CTCs, respectively.

Before the use of venous plasma C_p_(t) for Tofts model fitting, the venous CTC was shifted forwardly in time based on the peak time difference to account for the time delay between veins and arteries, accomplished in discrete steps given by the temporal resolution without interpolation because the acquisition temporal resolution was sufficiently high.

Primary tumors and metastatic nodes were identified and outlined on the anatomical MR images by a radiologist with over 15 years’ experience in head and neck MR imaging. Voxel-by-voxel fitting to Eq. 2 was performed for regions-of-interest (ROIs) of primary tumors as well as metastatic nodes within the central eleven inner slices from which the CTCs were estimated. Three independent parameters of k_ep_, K^trans^, and v_p_ were derived by non-negative least squares fitting based on Levenberg-Marquardt algorithm. The lower and upper limit of v_p_ for fitting were set as zero and one. The upper limit for k_ep_ fitting was set as three, sufficiently high to reveal the high vascularity of tumors and glands (such as salivary glands) in head and neck tissues. K^trans^ was constrained to be smaller than k_ep_. The sum of v_p_ and v_e_ were constrained to be smaller than one. Voxels that failed of fitting due to the violation of fitting restrictions or associated with the goodness of fit (R^2^) smaller than 0.8 were counted and excluded from the further analysis. Paired student’s t-test was performed to compare the fitting results by using the CTCs in arteries and veins. Consistency and variability of the extracted kinetic parameters by using the individual CTCs from each slice were compared to the reference levels by using the slice-averaged CTCs of arteries and veins with one-way analysis of variance (ANOVA) and Tukey–Kramer method. P-values less than 0.05 were considered statistically significant. Note that the reference defined here by using the slice-averaged CTCs were only used as the comparison baseline but did not necessarily indicate the gold standard for higher accuracy of parameter estimation.

## References

[1] Padhani AR (2002) Dynamic contrast-enhanced MRI in clinical oncology: current status and future directions. J Magn Reson Imaging 16: 407–422. 12353256 10.1002/jmri.10176

[2] Zahra MA, Hollingsworth KG, Sala E, Lomas DJ, Tan LT (2007) Dynamic contrast-enhanced MRI as a predictor of tumour response to radiotherapy. Lancet Oncol 8: 63–74. 17196512 10.1016/S1470-2045(06)71012-9

[3] O’Connor JP, Jackson A, Parker GJ, Roberts C, Jayson GC (2012) Dynamic contrast-enhanced MRI in clinical trials of antivascular therapies. Nat Rev Clin Oncol 9: 167–177. 22330689 10.1038/nrclinonc.2012.2

[4] Hoskin PJ, Saunders MI, Goodchild K, Powell ME, Taylor NJ, et al (1999) Dynamic contrast enhanced magnetic resonance scanning as a predictor of response to accelerated radiotherapy for advanced head and neck cancer. Br J Radiol 72: 1093–1098. 10700827 10.1259/bjr.72.863.10700827

[5] Sumi M, Nakamura T (2011) Extranodal spread in the neck: MRI detection on the basis of pixel-based time-signal intensity curve analysis. J Magn Reson Imaging 33: 830–838. 21448947 10.1002/jmri.22454

[6] Shukla-Dave A, Lee NY, Jansen JF, Thaler HT, Stambuk HE, et al (2012) Dynamic contrast-enhanced magnetic resonance imaging as a predictor of outcome in head-and-neck squamous cell carcinoma patients with nodal metastases. Int J Radiat Oncol Biol Phys 82: 1837–1844. 21601373 10.1016/j.ijrobp.2011.03.006 PMC3177034

[7] Noworolski SM, Fischbein NJ, Kaplan MJ, Lu Y, Nelson SJ, et al (2003) Challenges in dynamic contrast-enhanced MRI imaging of cervical lymph nodes to detect metastatic disease. J Magn Reson Imaging 17: 455–462. 12655585 10.1002/jmri.10280

[8] Donaldson SB, Betts G, Bonington SC, Homer JJ, Slevin NJ, et al (2011) Perfusion estimated with rapid dynamic contrast-enhanced magnetic resonance imaging correlates inversely with vascular endothelial growth factor expression and pimonidazole staining in head-and-neck cancer: a pilot study. Int J Radiat Oncol Biol Phys 81: 1176–1183. 21546171 10.1016/j.ijrobp.2010.09.039

[9] Kim S, Loevner LA, Quon H, Kilger A, Sherman E, et al (2010) Prediction of response to chemoradiation therapy in squamous cell carcinomas of the head and neck using dynamic contrast-enhanced MR imaging. AJNR Am J Neuroradiol 31: 262–268. 19797785 10.3174/ajnr.A1817 PMC7964131

[10] Bisdas S, Seitz O, Middendorp M, Chambron-Pinho N, Bisdas T, et al (2010) An exploratory pilot study into the association between microcirculatory parameters derived by MRI-based pharmacokinetic analysis and glucose utilization estimated by PET-CT imaging in head and neck cancer. Eur Radiol 20: 2358–2366. 20443116 10.1007/s00330-010-1803-x

[11] Brix G, Semmler W, Port R, Schad LR, Layer G, et al (1991) Pharmacokinetic parameters in CNS Gd-DTPA enhanced MR imaging. J Comput Assist Tomogr 15: 621–628. 2061479 10.1097/00004728-199107000-00018

[12] Tofts PS, Brix G, Buckley DL, Evelhoch JL, Henderson E, et al (1999) Estimating kinetic parameters from dynamic contrast-enhanced T1-weighted MRI of a diffusable tracer: standardized quantities and symbols. J Magn Reson Imaging 10: 223–232. 10508281 10.1002/(sici)1522-2586(199909)10:3<223::aid-jmri2>3.0.co;2-s

[13] Buckley DL (2002) Uncertainty in the analysis of tracer kinetics using dynamic contrast-enhanced T1-weighted MRI. Magn Reson Med 47: 601–606. 11870848 10.1002/mrm.10080

[14] Di Giovanni P, Azlan CA, Ahearn TS, Semple SI, Gilbert FJ, et al (2010) The accuracy of pharmacokinetic parameter measurement in DCE-MRI of the breast at 3 T. Phys Med Biol. 55: 121–132. 10.1088/0031-9155/55/1/008 20009182

[15] Evelhoch JL (1999) Key factors in the acquisition of contrast kinetic data for oncology. J Magn Reson Imaging 10: 254–259. 10508284 10.1002/(sici)1522-2586(199909)10:3<254::aid-jmri5>3.0.co;2-9

[16] Cheng HL (2007) T1 measurement of flowing blood and arterial input function determination for quantitative 3D T1-weighted DCE-MRI. J Magn Reson Imaging 25: 1073–1078. 17410576 10.1002/jmri.20898

[17] Roberts C, Little R, Watson Y, Zhao S, Buckley DL, et al (2011) The effect of blood inflow and B1-field inhomogeneity on measurement of the arterial input function in axial 3D spoiled gradient echo dynamic contrast-enhanced MRI. Magn Reson Med 65: 108–119. 20928889 10.1002/mrm.22593

[18] Hansen AE, Pedersen H, Rostrup E, Larsson HB (2009) Partial volume effect (PVE) on the arterial input function (AIF) in T1-weighted perfusion imaging and limitations of the multiplicative rescaling approach. Magn Reson Med 62: 1055–1059. 19672948 10.1002/mrm.22098

[19] Foottit C, Cron GO, Hogan MJ, Nguyen TB, Cameron I (2010) Determination of the venous output function from MR signal phase: feasibility for quantitative DCE-MRI in human brain. Magn Reson Med 63: 772–781. 20187184 10.1002/mrm.22253

[20] Lavini C, Verhoeff JJ (2010) Reproducibility of the gadolinium concentration measurements and of the fitting parameters of the vascular input function in the superior sagittal sinus in a patient population. Magn Reson Imaging 28: 1420–1430. 20817379 10.1016/j.mri.2010.06.017

[21] Ivancevic MK, Zimine I, Montet X, Hyacinthe JN, Lazeyras F, et al (2003) Inflow effect correction in fast gradient-echo perfusion imaging. Magn Reson Med 50: 885–891. 14586998 10.1002/mrm.10633

[22] Peeters F, Annet L, Hermoye L, Van Beers BE (2004) Inflow correction of hepatic perfusion measurements using T1-weighted, fast gradient-echo, contrast-enhanced MRI. Magn Reson Med 51: 710–717. 15065243 10.1002/mrm.20032

[23] Qin Q, Strouse JJ, van Zijl PC (2011) Fast measurement of blood T1 in the human jugular vein at 3 Tesla. Magn Reson Med 65: 1297–1304. 21500258 10.1002/mrm.22723 PMC3112465

[24] Holdsworth DW, Norley CJ, Frayne R, Steinman DA, Rutt BK (1999) Characterization of common carotid artery blood-flow waveforms in normal human subjects. Physiol Meas 20: 219–240. 10475577 10.1088/0967-3334/20/3/301

[25] Parker GJ, Roberts C, Macdonald A, Buonaccorsi GA, Cheung S, et al (2006) Experimentally-derived functional form for a population-averaged high-temporal-resolution arterial input function for dynamic contrast-enhanced MRI. Magn Reson Med 56: 993–1000. 17036301 10.1002/mrm.21066

[26] Schabel MC, Morrell GR (2009) Uncertainty in T1 mapping using the variable flip angle method with two flip angles. Phys Med Biol 54: N1–8. 19060359 10.1088/0031-9155/54/1/N01

[27] Yuan J, Chow SK, Yeung DK, Ahuja AT, King AD (2012) Quantitative evaluation of dual-flip-angle T1 mapping on DCE-MRI kinetic parameter estimation in head and neck. Quant Imaging Med Surg 2: 245–253. 23289084 10.3978/j.issn.2223-4292.2012.11.04 PMC3533599

[28] Leach MO, Morgan B, Tofts PS, Buckley DL, Huang W, et al (2012) Imaging vascular function for early stage clinical trials using dynamic contrast-enhanced magnetic resonance imaging. Eur Radiol 22: 1451–1464. 22562143 10.1007/s00330-012-2446-x

[29] Heywang-Kobrunner SH, Haustein J, Pohl C, Beck R, Lommatzsch B, et al (1994) Contrast-enhanced MR imaging of the breast: comparison of two different doses of gadopentetate dimeglumine. Radiology 191: 639–646. 8184040 10.1148/radiology.191.3.8184040

[30] Brookes JA, Redpath TW, Gilbert FJ, Needham G, Murray AD (1996) Measurement of spin-lattice relaxation times with FLASH for dynamic MRI of the breast. Br J Radiol 69: 206–214. 8800863 10.1259/0007-1285-69-819-206

[31] Yuan J, Chow SK, Yeung DK, King AD (2012) A five-colour colour-coded mapping method for DCE-MRI analysis of head and neck tumours. Clin Radiol 67: 216–223. 21939962 10.1016/j.crad.2011.07.052

[32] Yuan J, Chow SK, King AD, Yeung DK (2012) Heuristic linear mapping of physiological parameters in dynamic contrast-enhanced MRI without T1 measurement and contrast agent concentration. J Magn Reson Imaging 35: 916–925. 22095582 10.1002/jmri.22885

[33] Rijpkema M, Kaanders JH, Joosten FB, van der Kogel AJ, Heerschap A (2001) Method for quantitative mapping of dynamic MRI contrast agent uptake in human tumors. J Magn Reson Imaging 14: 457–463. 11599071 10.1002/jmri.1207

[34] Tofts PS (1997) Modeling tracer kinetics in dynamic Gd-DTPA MR imaging. J Magn Reson Imaging 7: 91–101. 9039598 10.1002/jmri.1880070113

[35] Noeske R, Seifert F, Rhein KH, Rinneberg H (2000) Human cardiac imaging at 3 T using phased array coils. Magn Reson Med 44: 978–982. 11108638 10.1002/1522-2594(200012)44:6<978::aid-mrm22>3.0.co;2-9

